# Sex Differences in Youth-Reported Depressive Symptomatology and Unwanted Internet Sexual Solicitation

**DOI:** 10.2196/jmir.6.1.e5

**Published:** 2004-02-06

**Authors:** Michele L Ybarra, Philip J Leaf, Marie Diener-West

**Affiliations:** ^1^Johns Hopkins Bloomberg School of Public HealthCenter for Adolescent HealthBaltimore MDUSA; ^2^Johns Hopkins Bloomberg School of Public HealthDepartment of Mental HygieneBaltimore MDUSA; ^3^Johns Hopkins Bloomberg School of Public HealthDepartment of BiostatisticsBaltimore MDUSA

**Keywords:** Youth, Internet, depression, sexual solicitation, mental health

## Abstract

**Background:**

As the number of youths using the Internet regularly increase, so too does the number of youths potentially vulnerable to negative experiences online. Clinicians, policy makers, and parents need to better understand the Internet and factors related to positive and negative experiences online.

**Objective:**

Primarily to investigate the association between youth-reported depressive symptomatology and unwanted Internet sexual solicitation and secondarily to identify sex differences in related characteristics of affected youth.

**Methods:**

Data from the Youth Internet Safety Survey were analyzed to investigate the association between reported depressive symptomatology and unwanted Internet sexual solicitation. The Youth Internet Safety Survey was a nationally-representative, cross-sectional telephone survey. Youth participants (N = 1501) were English speakers between the ages of 10 and 17 years who had accessed the Internet at least 6 times in the previous 6 months and had resided in the household for at least 2 weeks in the previous year. Eighty-two percent of contacted households agreed to participate.

Each participant was asked to indicate whether any of the 9 symptoms of major depression defined by the Diagnostic and Statistical Manual of Mental Disorders, Fourth Edition (DSM-IV) had been present within the previous month. Logistic regression was used to estimate the odds of reporting an unwanted sexual solicitation online for youths with mild or major depressive symptomatology versus no symptomatology. A parsimonious, multivariate model of significant youth characteristics was identified separately for males and females.

**Results:**

Youths who report major depressive-like symptoms were 3.5 times more likely (odds ratio, 3.54; 95% confidence interval, 2.19-5.71) to also report an unwanted sexual solicitation online compared to youths with mild/no symptomatology. After adjusting for significant Internet and psychosocial characteristics, male Internet users who report major depressive-like symptomatology were 2.5 times more likely to also indicate an unwanted Internet solicitation (adjusted odds ratio, 2.72; 95% CI, 1.15-6.40); significant differences were not observed among otherwise-similar females. Further, among youths reporting an Internet solicitation (N = 283), youths with major depressive-like symptomatology were twice as likely to report feeling emotionally distressed by the incident compared to youths with mild/no symptomatology (odds ratio, 2.27; 95% CI, 1.03-5.02).

**Conclusions:**

While the majority of youths report positive experiences online, some youths may be more vulnerable to negative experiences. Cross-sectional results indicate that the report of depressive symptomatology is significantly related to the concurrent report of an unwanted Internet sexual solicitation, especially for young males. Future research should focus on parsing out the temporality of events and identifying additional populations of vulnerable youths online.

## Introduction

An estimated 97% of youths between the ages of 12 and 18 use the Internet in the United States [1]. Although youths consistently report positive aspects of Internet use [2- 4], young people, parents, and politicians have raised concerns about the possible deleterious effects of exposure to negative sexual experiences online, including sexual material and pedophiles [2,5- 6]. Given the almost complete saturation of Internet use among children and adolescents, research about the experiences young people are having online and their effect on somatic and mental health is needed.

Unwanted online sexual solicitation is one such online experience that may affect the health and functioning of a young person. It occurs when a youth is encouraged to engage in sexual activity when he or she does not wish to. Specifically, it is being overtly persuaded to talk about sex with someone, doing something sexual, or disclosing personal sexual information when not wanted [2,7,8]. This may occur as part of "preening," during which an adult starts a nonsexual relationship with a child online to build trust and then segues into seducing the child into sexual acts [6]. It may also occur in a peer-to-peer exchange between 2 young people of similar age who are communicating online.

O'Connell (2003) [9] has conducted a qualitative analysis of online chat exchanges between herself, posing as a young girl, and predatory adults. Data were collected over 5 years. Findings reveal the truly-threatening aspects of online sexual solicitation for some young people. The data suggest that emotionally-vulnerable and socially-disconnected youths may be at greater risk for pedophilic targeting. The selection bias inherent in nonrandomly-selected case studies however, disallows inference and generalization to the online youth population, or comparisons between solicited and nonsolicited young people-both of which are necessary to inform future prevention and intervention research.

Berson et al [10] conducted a survey of adolescent girls who use the Internet in the effort to begin understanding their experiences of sexual solicitation online. The Web-based study included recruitment messages on Seventeen Magazine Online. Ten thousand eight hundred youths completed the online survey. Findings suggest that the anonymous nature of online exchanges may lead to wrong conclusions about the level of risk or safety an online acquaintance may represent. Indeed, some people prefer Internet-based relationships given the ease of deception this type of communication allows. While not all people who enjoy this anonymity have ill intent, there are those who take advantage of the inscrutability to prey on susceptible young people. Berson [11] notes that, although intimate and positive relationships are formed online by young people, the culture of "deception" in cyberspace conversations is a reason for concern with respect to vulnerable adolescent females. These results justify further examination of online sexual solicitation of youths, preferably with a randomly selected group of young people to allow the report of prevalence rates and comparisons of related characteristics.

A recent, nationally-representative telephone survey of youths in the United States between the ages of 12 and 17 indicates that 1 in 5 youths have been the target of an unwanted sexual solicitation within the previous year [12]. Personal characteristics associated with unwanted sexual solicitation include being female, older (ie, between 14 and 17 years of age), and psychosocially challenged [12]. Although most youths reporting an unwanted sexual solicitation also report little emotional harm, 25% reveal experiencing extreme emotional distress [12].

Despite the broad-ranging interest in exposure to sexual experiences among youths [2,6] , and indication that unwanted sexual solicitation is associated with emotional distress for one-quarter of those targeted [12], we know little about subpopulations that may be more vulnerable to sexual solicitation online. For example, there is a noticeable lack of information about how these experiences affect young people with mental health problems such as depression. Major depressive disorder affects an estimated 2% to 8% of youths at any given time [13]. There is a strong literature base that indicates traumatic events can lead to depressive symptomatology for children and adolescents [14]. It is possible then, that a sexual solicitation will be related to a subsequent onset of a major depressive episode. It is alternatively possible that young people with major depressive symptomatology are more susceptible to online solicitations [15]. Depressive symptomatology is in fact related to risk for unhealthy sexual experiences, specifically sexual abuse [16] and risky sexual behavior [17]. Given the significant personal distress and public health burden that is related to child and adolescent depression [18], it is vitally important that we begin to understand how unwanted sexual solicitation is related to this disorder. Establishing a baseline association between depressive symptomatology and unwanted sexual solicitation is a necessary first step in parsing out the relationship between mental health and the important public health phenomenon, Internet sexual solicitation.

### Gap in Current Literature

Despite the extensive numbers of young people who are immersed in the Internet culture, researchers and clinicians continue to be disadvantaged by the paucity of research based upon representative samples of young people that analyze the interplay between psychosocial functioning and experiences youths have online. To begin addressing this gap, the current research will examine cross-sectional data to identify trends in the relationship between youth-reported depressive symptomatology and unwanted Internet sexual solicitation. We then examine gender-specific analyses to identify possible differences by sex as hypothesized based upon the reported differences in prevalence rates of depressive symptomatology [19,20] and sexual abuse [21,22]. Internet usage and psychosocial characteristics are examined for potential confounding of the observed relationships. Finally, the association between depressive symptomatology and self-reported sequelae of the unwanted sexual solicitation is estimated.

## Methods

The Youth Internet Safety Survey (YISS) was a nationally-representative telephone survey of young, regular Internet users, and one caregiver in the household focusing on youth Internet harassment, unwanted sexual solicitation, and unwanted exposure to sexual material. Use of the YISS data was provided by Dr. David Finkelhor and colleagues at the University of New Hampshire Crimes Against Children Research Center. The cross-sectional survey was conducted between fall 1999 and spring 2000. Approved and supervised by the University of New Hampshire's Human Subjects Committee, the YISS was commissioned by the National Center for Missing and Exploited Children, supported by the Office of Juvenile Justice and Delinquency Prevention, and conformed to the Department of Justice's rules for research projects funded by the agency. Approval to perform the current data analyses was obtained by the Johns Hopkins Bloomberg School of Public Health's Committee for Human Research.

### Study Population

Youth participants (N = 1501) were English speakers between the ages of 10 and 17 years who had accessed the Internet at least 6 times in the previous 6 months. Location of Internet access was broadly defined to include all possible locations (eg, home, library, another person's home) to ensure a variety of Internet users reflective of the general population of users. Additionally, youths were required to have resided in the household for at least 2 weeks in the previous year. The caregiver who was interviewed was self-identified as the one most knowledgeable about the youth's Internet activity. Informed consent from the caregiver was required before the commencement of his or her interview, and both caregiver and youth consented before the youth interview.

The sample characteristics have been reported previously [7,12]. The average age of respondents was 14.15 years. Fifty-two percent of youth respondents were male, and 69% of adult respondents were female. Three quarters of youth participants self-identified as white, with an additional 10% self-identifying as black. Seven percent of youth respondents self-identified as being of Hispanic ethnicity. One quarter of households reported a 1999 income of $75000 or higher and more than 1 in 5 reported the highest household education as being at least some college. These household characteristics were higher than the United States average [23], but were consistent with reports of households with Internet connections at the time of data collection [24,25].

### Design and Procedures

Details of YISS sampling methods have been detailed elsewhere [12]. Phone numbers were first generated for the Second National Incidence Study of Missing, Abducted, Runaway, and Thrownaway Children [26], a nationally-representative telephone survey targeting United States youth. Phone numbers for households that were identified during their initial screening process as having at least 1 youth residing who was between the ages of 9 and 18 were forwarded to YISS researchers for future contact. A sample size of 1500 youth respondents was targeted before YISS data collection, in order to achieve a sampling error of ±2.5% at the 5% significance level. Of the 72% of households that were contacted and eligible (N = 1857), 82% (N = 1501) completed both the youth and caregiver surveys.

### Measures

#### Unwanted Sexual Solicitation

Three questions used in earlier investigations [7,12] were used to identify youths who had been sexually solicited online in the previous year: (1) whether anyone had asked the youth to talk about sex when he or she did not want to; (2) whether the youth had been asked to disclose personal, sexual information, such as sexual experiences or body type, about him or herself; and (3) whether anyone had asked the youth to do something sexual that he or she did not want to. Respondents who endorsed at least 1 of the 3 questions, as well as all solicitations that involved an adult (whether deemed "wanted" or "unwanted" by the young person) were considered to have been exposed to an unwanted sexual solicitation.

#### Depressive Symptomatology

Youths were asked 9 dichotomous (yes/no) questions based upon the Diagnostic and Statistical Manual of Mental Disorders, Fourth Edition (DSM-IV) definition of major depressive disorder [27]. Each referred to any time within the last month except for dysphoria, which referred to most of the day, nearly every day for the previous 2 weeks. Reflective of the DSM-IV criteria for major depression, 3 additional questions were asked about functional challenge, including self-efficacy, personal-hygiene, and schoolwork. Respondents were categorized into 1 of 3 categories: (1) major depressive-like symptomatology (ie, 5 or more symptoms, 1 of which was anhedonia or dysphoria, and functional impairment in at least 1 area); (2) minor depressive-like symptomatology (ie, at least 3 symptoms of depression); or (3) no/mild symptomatology (fewer than 3 symptoms).

#### Internet Usage Characteristics

Exploratory factor analysis identified a latent variable described as "interactive Internet activity." Each eigenvalue was greater than or equal to 1. Eigenvalues are commonly used as a useful tool to identify factors because they are related to the underlying factor's ability to explain the correlations between the observed variables included in the exploratory factor analysis. An eigenvalue of 1 is a widely-accepted cutoff when identifying factors. The variables included in this factor were:

- using the Internet (ever) for Instant messaging, e-mailing, downloading files, updating a Web page, connecting to a news group, visiting chat rooms, and looking up movie information
- logging onto the Internet from home versus all other places
- using the Internet 5 or more days a week
- self-rated Internet expert (almost or definitely) versus being less skilled
- importance of Internet to self (very, extremely) versus less importance.

Factor scores were used to categorize respondents into 1 of 3 groups:

1. highly interactive (1 or more SD above the mean)
2. average interactive (scores within 1 SD of the mean)
3. less than average (1 or more SD below the mean; reference group).

In addition, youths were asked to identify the activity for which they used the Internet most. This activity was grouped into 4 categories based upon frequency and speed of interaction:

1. chat rooms
2. e-mail
3. instant messaging
4. all else (reference group).

Finally, youths who reported online aggression towards another person were identified by their engaging in at least 1 of 2 Internet harassment activities:

1. making rude or nasty comments to someone else online
2. harassing or embarrassing someone else on the Internet.

#### Substance Use

Youth respondents were asked about the frequency of 5 types of substance use in the previous year, including: tobacco, alcohol, inhalants, marijuana, and all other drugs. Each was dichotomized (4 or more times versus fewer) to put the variables on the same scale as other variables included in the exploratory factor analysis. One factor was identified (eigenvalue greater than or equal to 1), which included all 5 variables. Because of the data distribution of the sum of the 5 variables, total scores were categorized into 3 groups:

1. low users (1 or more SD below the mean; reference group)
2. average users (scores within 1 SD of the mean)
3. heavy users (1 or more SD above the mean).

#### Life Challenge

Indication of life challenge was also included because of its association with depressive symptoms [28]. Thus, interpersonal challenge was noted for young people who reported 2 or more versus fewer of the following events:

- being attacked by 1 person
- being attacked by a gang
- having something stolen from the young people
- being hit by a peer
- by being "picked on" by a peer in the previous year.

Further, 2 or more life challenges (range, 0-4) in the previous year included the following experiences:

- death in the immediate family
- moving to a new community
- caregiver divorce
- loss of job among the caregivers in the previous year.

### Data Analysis

For the purposes of the current investigation, cases included in the analyses were required to have valid data for the majority of variables assessed. Records missing more than 2 variables within a subcategory of analytic interest (ie, depressive symptomatology, Internet use, demographics, negative life experiences, substance use), or those missing 2 variables across 2 or more subcategories, were dropped. Nine cases met these criteria and were excluded from further analyses. Additionally, factor scores could not be estimated for 3 cases because of unstable estimates, resulting in a final sample size of 1498 youth.

Missing values were imputed to maximize available data using best-set regression techniques [29]. Values were estimated based upon responses for depressive symptoms, unwanted Internet experiences (ie, sexual solicitation and Internet harassment), race, age, and sex; in most cases, this involved less than 1% of cases. "Don't know" and "refused" responses were coded at the variable mean, and therefore most often as "symptom absent." In most instances, this affected less than 1% of all cases. Finally, 3 separate "dummy" variables were created to reflect whether data for each case had been manipulated by (1) imputation, (2) recoding for "don't know," or (3) recoding for "refused" response.

MPlus was used to estimate several hypothesized latent variables [30]. All variables were included in 1 exploratory factor analysis to adjust for unanticipated cross correlations across hypothesized factors. The final factor solution was identified based upon the combination of eigenvalues, scree plots, and root mean square residuals. Scores were estimated using varimax rotation. Seven factors were identified: Internet use, substance use, depressive symptomatology, and 4 aspects of the caregiver-child relationship (not included in the current analyses).

Using Stata 7 [29], bivariate relationships between variables were assessed using the chi-square statistic. Logistic regression modeling was used to estimate the odds of reporting an unwanted sexual solicitation online for youths who report major or minor depressive-like symptomatology versus mild/no symptomatology. Upon observation that the association between depressive symptomatology and Internet sexual solicitation was different for males and females, the sample was stratified and a parsimonious logistic regression model of significant factors was developed separately for each sex. Two additional youth characteristics were investigated for effect modification: substance use [15,31] and age [32,33].

A saturated logistic regression model, including all youth characteristics and interaction terms, was first fit. Variables were then deleted based upon backward stepwise and forward stepwise tests for significance ( *P*< .1). Variables identified in either stepwise solution were then entered into one model and tested for significant contribution to the overall model based upon likelihood ratio tests ( *P*< .05). Depressive symptomatology and the 3 dummy variables (ie, indication of a refused response, don't know response, and imputed variable) were retained in the final model regardless of significance. Each final model was tested for goodness of fit using the Hosmer-Lemeshow goodness-of-fit test ( *P*< .5 indicates better fit). All regression analyses were performed using the Stata statistical analysis package [29].

## Results

### Descriptive Results

In the current study, 12% (N = 94) of male and 27% (N = 189) of female young regular Internet users reported at least 1 unwanted sexual solicitation in the previous year. Five percent (N = 77) of young regular Internet users met criteria for major depressive-like symptomatology in the previous month. An additional 14% (N = 211) met criteria for minor depressive-like symptoms. Females were 60% more likely (OR = 1.59; 95% CI, 1.00-2.54) (OR = odds ratio, CI = confidence interval) to report major depressive-like symptomatology compared to males, although no sex differences were observed for youths with minor depressive-like symptomatology versus no depressive symptoms (OR = 0.91; 95% CI, 0.68-1.22). The odds of major depression-like symptomatology increased 19% with each year in age of the respondent (OR = 1.19; 95% CI, 1.05-1.35), while no significant differences in the odds of minor depressive-like symptomatology were indicated by age (OR = 1.0; 95% CI, 0.92-1.07).

Eleven percent of youths reporting an unwanted online sexual solicitation also reported major depressive-like symptomatology as compared to 3.7% of youths who were not solicited (χ ^2^_1_= 26.8, *P*< .001). Similarly, 17.7% of youths who indicated sexual solicitation online also reported minor depressive-like symptoms compared to 13.4% of youths indicating no solicitation event (χ ^2^_1_= 3.5, *P*= .06). Further, among youths who report an unwanted sexual solicitation online (N = 283), those meeting criteria for major depressive-like symptomatology were more than twice as likely (OR = 2.27; 95% CI, 1.03-5.02) to indicate they felt emotionally distressed by the incident compared to youths with mild/no symptoms of depression.

### Reports From Male Internet Users

The most parsimonious logistic regression model of significant factors related to the report of unwanted sexual solicitation online for males (N = 782) is found in Table 1. Acceptable model fit was indicated for the parsimonious model (Hosmer-Lemeshow goodness of fit χ ^2^_7_= 1.6, *P*= .98). Effect modifications of the relationship between solicitation and depressive symptomatology by substance use and age were explored but resulted in unstable estimates and were not included in the steps for building the final model. Final estimates were adjusted for the effects of "don't know," refused, and imputed responses; however, these did not differ significantly from the unadjusted estimates.

**Table 1:** Final logistic regression model of significant characteristics associated with unwanted Internet sexual solicitation for Male Internet users (N = 782)

**Male Youth Characteristics**			Adjusted Odds Ratio* (95% Confidence Interval)	**P**
**Depressive symptoms**				
	Symptoms of major depression		2.72 (1.15, 6.40)	.02
	Symptoms of minor depression		0.89 (0.45, 1.77)	.74
	Mild/Absent symptoms		1.00 (reference group)	
**Internet usage characteristics**				
	Interactive Internet use			
		Frequent	4.80 (2.47, 9.35)	< .001
		Moderate	2.13 (1.16, 3.94)	.02
		Infrequent	1.00 (reference group)	
	Most frequent Internet activity			
		Chat room	3.13 (1.60, 6.11)	< .001
		E-mail	1.57 (0.84, 2.94)	.16
		Instant Messaging	1.10 (0.52, 2.32)	.80
		All other	1.00 (reference group)	
	Harasser of others online		1.80 (1.01, 3.20)	.05
**Psychosocial characteristics**				
	Life challenge (≥ 2)		2.94 (1.33, 6.50)	.01
	Interpersonal victimization (≥ 2)		1.87 (1.12, 3.14)	.02

^*^ Adjusted odds ratio = the odds ratio estimated after adjusting for all other variables included in the parsimonious model.

Males who reported symptoms of major depression were almost 6 times as likely (OR = 5.90; 95% CI, 2.79-12.49) to report an unwanted Internet sexual solicitation compared to males indicating mild/absent symptoms of depression. No significant differences were observed however, between males who indicated minor depressive-like symptomatology, and mild/absent symptoms of depression (OR = 1.29; 95% CI, 0.71-2.36). After adjusting for all other significant characteristics, males who reported symptoms of major depression were almost 3 times as likely (adjusted OR = 2.72; 95% CI, 1.15-6.18) to also report an unwanted sexual solicitation compared to otherwise-similar males who reported mild/absent symptoms of depression (see Table 1). (Adjusted odds ratio is the odds ratio estimated after adjusting for all other variables included in the parsimonious model.)

In addition to depressive symptomatology, interactive Internet activity, using the Internet most frequently for logging onto chat rooms, and harassing others online were associated with increased odds of reporting an unwanted Internet sexual solicitation among males after adjusting for other significant characteristics (see Table 1). Multiple psychosocial indicators, including life challenge and interpersonal victimization also were significantly related to the odds of reporting an unwanted sexual solicitation for males.

### Reports From Female Internet Users

A parsimonious logistic regression model of significant factors related to the report of unwanted sexual solicitation online for females (N = 707) is in Table 2. Acceptable model fit was indicated for the final logistic regression model (Hosmer-Lemeshow goodness of fit χ ^2^_204_= 6.4, *P*= .60). We were unable to determine whether substance use and age affected these relationships because analyses produced unstable estimates. The final model estimates were adjusted for the effects of don't know, refused, and imputed responses, although unadjusted results did not differ. Young regular Internet-using females who reported symptoms of major depression were twice as likely to also report an unwanted sexual solicitation online (OR = 2.33; 95% CI, 1.25-4.35), while females who reported symptoms of minor depression were 80% more likely (OR = 1.83; 95% CI, 1.16-2.90) to indicate an Internet event compared to females indicating mild/absent symptoms of depression. After controlling for other significant characteristics, a trend for increased odds of reporting Internet sexual solicitation was still observed for females reporting minor or major depressive symptoms, although it was no longer statistically significant.

**Table 2:** Final parsimonious model of significant characteristics associated with unwanted Internet sexual solicitation for female Internet users (N = 707)

**Female Youth Characteristics**			**Adjusted Odds Ratio** **(95% Confidence Interval)**	**P**
**Depressive symptoms**				
	Symptoms of major depression		1.40 (0.65, 2.99)	.39
	Symptoms of minor depression		1.62 (0.96, 2.76)	.07
	Mild/Absent symptoms		1.00 (reference group)	
**Internet usage characteristics**				
	Harasser of others online		4.07 (2.48, 6.68)	< .001
	Interactive Internet use			
		High	3.21 (1.79, 5.77)	< .001
		Moderate	2.12 (1.34, 3.37)	< .001
		Infrequent	1.00 (reference group)	
	Most frequent Internet activity			
		Chat room	3.10 (1.62, 5.93)	< .001
		Instant Messaging	1.34 (0.68, 2.62)	.39
		E-mail	1.30 (0.81, 2.07)	.28
		All other	1.00 (reference group)	
**Psychosocial characteristics**				
	Substance use			
		High user	2.87 (1.13, 7.34)	.03
		Average user	2.09 (0.97, 4.53)	0.06
		Mild/non-user	1.00 (reference group)	
	Interpersonal victimization (≥ 2)		1.82 (1.15, 2.89)	0.01

Multiple Internet usage characteristics, including harassing others, using the Internet for interactive Internet activities, and using the Internet most frequently for chat room use, were related to the likelihood of reporting an unwanted sexual solicitation for female Internet users after adjusting for other influential factors (see Table 2). Substance use and interpersonal victimization were also related to significantly higher odds of also reporting an unwanted sexual solicitation online among otherwise similar female Internet users.

Additional covariates that were tested but found not to be significantly associated with the report of sexual solicitation online after adjusting for depressive symptomatology included: type of Internet service provider, average daily Internet usage, sexual or physical abuse, the number of close friends, the number of times a youth spent time with friends outside of school in a typical week, household income, age, and race and ethnicity. Also, separate models for each of the 3 outcomes of unwanted Internet sexual solicitation, ie, being encouraged to discuss sexual topics, being encouraged to engage in sexual behavior, and being encouraged to divulge personal sexual information, were constructed to check for consistency of association with reported depressive symptomatology. In each model, depressive symptomatology was related to significantly greater odds of unwanted solicitation, thus supporting the use of the combined solicitation outcome in our analyses.

## Discussion

Unwanted Internet sexual solicitation is reported frequently by young regular Internet users. Almost 1 in 3 females (27%) and 1 in 10 males (12%) indicate they have been approached in the previous year. These cross-sectional results indicate a trend for self-reported depressive symptomatology to be associated with the concurrent odds of indicating an online solicitation. Findings suggest that the report of symptoms of major depression is associated with a 3-fold increase in odds of concurrently reporting an event. Sex-specific differences are noted, revealing that this association is especially strong for males. Males who report major depressive symptoms are almost 6 times as likely to also indicate an unwanted sexual solicitation compared to males reporting mild/absent symptoms of depression after adjusting for significant confounders. On the other hand, females reporting major depressive symptoms are twice as likely to indicate an unwanted sexual solicitation versus females reporting mild/absent symptoms.

Underlying differences in Internet use and psychosocial challenge explain the association between depressive symptomatology and the unwanted Internet solicitation among females. Among otherwise-similar males however, a continued association between major depressive symptomatology and unwanted sexual solicitation is observed. This is especially intriguing, for it implies that differences in Internet usage (eg, frequenting chat rooms), do not sufficiently explain variation in the odds of reporting an unwanted online sexual solicitation for males. These cross-sectional results raise an intriguing question for future research: are males more likely to experience depressive symptomatology because of the event, or is it that males who are experiencing depressive symptomatology are more likely to be targeted, or more probably, is the relationship bidirectional?

The especially-strong association between depressive symptomatology and Internet sexual solicitation among males may be because they are less prepared to handle an advance. More than twice as many females are the target of unwanted sexual solicitation, and it is possible that some male victims see themselves as being singled out for something that "should" be directed to females. It may be also that males receive less emotional support following the event, either because they are reticent to talk about it with potentially-ridiculing peers, or because others dismiss the emotional impact of the event, assuming that it must be less upsetting or even gratifying for the male. In either scenario, the sexual solicitation is experienced as a traumatic event for males and may be a contributing factor for subsequent depressive symptomatology. Irrespective of temporality, these results indicate that health professionals need to be especially attuned to the Internet experiences of young men with depressive symptomatology.

### Psychosocial Challenge

Psychosocial challenge is associated with the report of unwanted sexual solicitation online. Beyond major depressive-like symptomatology, results suggest that multiple life challenges and interpersonal victimization are each concurrently related to the report of unwanted Internet sexual solicitation for males. Females who report unwanted sexual solicitation also indicate psychosocial challenge; specifically, high substance use and interpersonal victimization are each associated with elevated odds of reporting an unwanted sexual solicitation. Thus, a youth's indication of multiple mental health-related problems is associated with also reporting sexual solicitation online for both sexes. Future research should focus on understanding why depressive symptomatology is part of the picture of challenge for males but not for females.

Drawing upon theories of depression, the profile of multiple and varied victimization experiences may reflect a type of "learned helplessness" [34] for boys who are manifesting depressive symptomatology. They may be less likely to be assertive and self-protective, thereby increasing the chances of exploitation. If true, the consequence appears to be multiple victimizations, including Internet sexual solicitation and offline bullying.

### Emotional Distress

Although a cross-sectional study, youths who reported an unwanted sexual solicitation were queried about how they felt as a direct result of the event, thereby allowing temporal inference. One quarter of youths who report online solicitations indicate they feel very or extremely upset or afraid as a result [7]. The current investigation indicates that the report of depressive symptomatology is associated with 2-fold increased odds of reporting emotional distress due to the solicitation incident for youths who indicate major depressive-like symptoms compared to youths with mild/no symptoms. Given that the accumulation of negative life events is related to an increased risk of the onset of a major depressive episode among youths [14], it is possible that this negative experience will be related to an increased risk for subsequent depressive symptoms. It is also possible that youths with depressive symptoms are not only more likely to be solicited online, but also to be emotionally affected by the incident. Future longitudinal studies are needed to further understand this important association.

### Cognitive Distortion

Some may conjecture that the observed relationship between reported depressive symptomatology and indications of sexual solicitation are perhaps a matter of cognitive distortion. Youths with depressive symptomatology are generally more likely to cognitively bias their perceptions of events in a negative manner [35]. Their perception of events may be skewed such that, what other young people view as neutral or mildly annoying, young people with depressive symptoms perceive as threatening or emotionally upsetting. Thus, youths indicating symptoms of depression are more likely to report an unwanted sexual solicitation simply because they are more likely to perceive an interaction as solicitous and personally threatening. Differences in interpretation and reaction to online events are certainly areas for future research; the current data do not allow disentanglement. What is revealed from the current results however, is that, whether due to cognitive distortion or not, almost 40% of young people who report major depressive symptomatology also report feeling very or extremely upset or afraid as a direct result of an unwanted sexual solicitation compared to 21% of young people who report mild/absent symptoms of depression ( *P*< .05). Further, recent research about the grooming of children for cyber-victimization [9,10] indicates that it is vitally important that online sexual solicitation be neither marginalized nor viewed as a contrived problem. Transcripts from online dialogues convey the truly-threatening aspects of some of these interchanges [9]. The fact that depressive symptomatology and unwanted sexual solicitation are cross-sectionally related reveals an emerging public health and mental health issue that warrants further investigation.

### Pedophilia or Peer-to-Peer Sexual Solicitation

The majority of research about online sexual solicitation has focused on adult-to-child solicitation and predation [9,11,36]. Although this is clearly an important health topic, this should not overshadow the fact that adolescent peers are soliciting each other. Almost half (48%) of YISS respondents who indicate they have been targeted for unwanted sexual solicitation report that the solicitor is less than 18 years of age [12]. About one quarter (24%) of solicitors are reported by the targeted YISS respondent to be over 18 years of age, and the remaining 27% of incidents involve a solicitor of unknown age. It is possible that an adult posing as a child, thus leading the young person to believe the solicitor was less than 18 years of age, is the real perpetrator in some of these incidents. It is also possible that peers carry out unwanted sexual solicitation on each other. Just as adolescents commit traditional sexual assault on their peers, it is likely that adolescents perpetrate unwanted sexual solicitation online towards their peers. This type of victimization must be acknowledged in Internet-based sexual solicitation discourse and must be discussed in intervention or prevention materials aimed at young people.

### Limitations

Although the YISS is the largest, most detailed survey of young regular Internet users to date, it is not without limitations. First, the cross-sectional nature of the study precludes inferences about temporality. Nothing can be inferred regarding the causal relationship between depressive symptoms and unwanted sexual solicitation. This important baseline association however, justifies future research endeavors aimed at parsing out the relationship between depression and Internet sexual solicitation, as well as youth-oriented prevention programs. Second, a validated scale for major depressive disorder (MDD) was not used to measure symptoms. Because the variables were based directly upon the DSM-IV criteria for major depressive disorder, it can be concluded that youths are experiencing depressive symptomatology, but they do not necessarily meet the criteria for disorder. Third, it is possible that the report of sexual solicitation was underreported, or that the severity of the experience was downplayed by the adolescent because he or she was concerned that others might be listening to the conversation. Researchers were aware of this possibility however, and encouraged youths to identify a time to conduct the interview when they would feel comfortable to disclose private and personal information [12]. All attempts were made to ensure the youth was in a private environment. Interviewers offered to call back at a more convenient time if the respondent was concerned about confidentiality. Finally, the exclusion of non-English speaking youths disallows the generalization to youths of other cultures.

### Implications

Despite limitations, the current investigation contributes to the knowledge base of youth-related mental health issues and Internet experiences. For youths who indicate major depressive-like symptomatology, we found an increased likelihood of also reporting an unwanted sexual solicitation online. Further, these youths are likely experiencing multiple challenges, including negative life experiences and substance use. It is imperative that health professionals, policy makers, and parents are well versed on the activities youths are engaging in online; this seems to be especially true for mental health practitioners given the multitude of personal challenges reported. Future studies should also look at the association between mental health and susceptibility to "preening," a more-subtle yet dangerous form of online sexual solicitation [6].

### Conclusions

The current study examines cross-sectional data to provide the necessary initial report of associations and trends to justify future research. Findings suggest that for males, differences in Internet usage alone are not sufficient to explain the odds of reporting an unwanted online sexual solicitation. Further, young people reporting solicitation online are likely experiencing significant psychosocial challenge, including depressive symptomatology for males, interpersonal victimization, and substance use for females. Additional education about the Internet is necessary to ensure youths affected by negative online experiences are appropriately identified.

The Internet is a pervasive mode of peer interaction in the lives of young people today. The linkages between online experiences and mental health, as both conferred vulnerability as well as related sequelae, must therefore be foremost in the minds of child public health researchers and providers. Understanding the complex interaction between mental health and online interactions, especially the influence of malleable characteristics such as Internet usage and psychosocial challenge, is an important area of emerging research.

## Abbreviations

CI: confidence interval
DSM-IV: Diagnostic and Statistical Manual of Mental Disorders, Fourth Edition
OR: odds ratio
YISS: Youth Internet Safety Survey

## References

[1] UCLA Center for Communication Policy (2003). UCLA Internet Report: Year Three.

[2] Lenhart Amanada (2001). Teenage life online: The rise of the instant-message generation and the Internet's impact on friendships and family relationships.

[3] Borzekowski D L, Rickert V I (2001). Adolescent cybersurfing for health information: a new resource that crosses barriers. Arch Pediatr Adolesc Med.

[4] Rideout V Generation Rx.com: how young people use the Internet for health information. A Kaiser Family Foundation survey.

[5] Federal Trade Commission (1999). Children's online privacy protection rule; final rule. Federal Register.

[6] Deirmenjian John M (2002). Pedophilia on the Internet. J Forensic Sci.

[7] Mitchell K J, Finkelhor D, Wolak J (2001). Risk factors for and impact of online sexual solicitation of youth. JAMA.

[8] Stahl Christiane, Fritz Nancy (2002). Internet safety: adolescents' self-report. J Adolesc Health.

[9] O'connell R (2003). A typology of child cybersexploitation and online grooming practices.

[10] Berson IR, Ferron JM (2002). Emerging risks of violence in the digital age: lessons for educators from an online study of adolescent girls in the United States. J School Violence.

[11] Berson IR (2003). Grooming cybervictims: the psychosocial effects of online exploitation for youth. J School Violence.

[12] Finkelhor D, Mitchell KJ, Wolak J (2000). Online victimization: a report on the nation's youth.

[13] Kessler R C, Walters E E (1998). Epidemiology of DSM-III-R major depression and minor depression among adolescents and young adults in the National Comorbidity Survey. Depress Anxiety.

[14] Cohen LH, Burt CE, Bjorck JP (1987). Life stress and adjustment: effects of life events experienced by young adolescents and their parents. Dev Psychol.

[15] Lewinsohn P M, Hops H, Roberts R E, Seeley J R, Andrews J A (1993). Adolescent psychopathology: I. Prevalence and incidence of depression and other DSM-III-R disorders in high school students. J Abnorm Psychol.

[16] Boney-mccoy S, Finkelhor D (1996). Is youth victimization related to trauma symptoms and depression after controlling for prior symptoms and family relationships? A longitudinal, prospective study. J Consult Clin Psychol.

[17] Shrier L A, Harris S K, Sternberg M, Beardslee W R (2001). Associations of depression, self-esteem, and substance use with sexual risk among adolescents. Prev Med.

[18] US Department of Health and Human Services (1999). Mental health culture, race, and ethnicity : a supplement to Mental health, a report of the Surgeon General : executive summary (SuDoc HE 20.402:M 52/2/SUPP./EXEC.SUM./2001).

[19] Cole David A, Tram Jane M, Martin Joan M, Hoffman Kit B, Ruiz Mark D, Jacquez Farrah M, Maschman Tracy L (2002). Individual differences in the emergence of depressive symptoms in children and adolescents: a longitudinal investigation of parent and child reports. J Abnorm Psychol.

[20] Rushton Jerry L, Forcier Michelle, Schectman Robin M (2002). Epidemiology of depressive symptoms in the National Longitudinal Study of Adolescent Health. J Am Acad Child Adolesc Psychiatry.

[21] Nolen-hoeksema S, Girgus J S (1994). The emergence of gender differences in depression during adolescence. Psychol Bull.

[22] Feiring C, Taska L, Lewis M (1999). Age and gender differences in children's and adolescents' adaptation to sexual abuse. Child Abuse Negl.

[23] US Census Bureau United States Census 2000.

[24] National Public Radio, Kaiser Family Foundation, Kennedy School of Government (2000). National survey of American adults on technology / National survey of American kids on technology.

[25] Lebo H (2001). The UCLA Internet report 2001 - Surveying the digital future: year two.

[26] Hammer H, Finkelhor D, Sedlak AJ (2002). NISMART-2 household survey methodology technical report.

[27] Association American Psychiatric (1994). Diagnostic and Statistical Manual of Mental Disorders DSM-IV.

[28] Kazdin AE, Marciano PL, Mash Eric J, Barkley Russell A (1998). Childhood and adolescent depression. Treatment of Childhood Disorders.

[29] Stata Corp Stata Statistical Software [computer program].

[30] Muthen Linda K (1998). Mplus: Statistical analysis with latent variables : user's guide.

[31] Anderson J C, Williams S, Mcgee R, Silva P A (1987). DSM-III disorders in preadolescent children. Prevalence in a large sample from the general population. Arch Gen Psychiatry.

[32] Carlson G A, Kashani J H (1988). Phenomenology of major depression from childhood through adulthood: analysis of three studies. Am J Psychiatry.

[33] Fleming J E, Offord D R (1990). Epidemiology of childhood depressive disorders: a critical review. J Am Acad Child Adolesc Psychiatry.

[34] Abramson L Y, Seligman M E, Teasdale J D (1978). Learned helplessness in humans: critique and reformulation. J Abnorm Psychol.

[35] Quiggle N L, Garber J, Panak W F, Dodge K A (1992). Social information processing in aggressive and depressed children. Child Dev.

[36] O'connell R (2001). Be somebody else but be yourself at all times: degrees of indentity deception in chatrooms.

